# Improving Ways to Generate and Use Local Data to Create and Strengthen Binational Programs

**Published:** 2008-09-15

**Authors:** Jorge S Hernández

**Affiliations:** Health Quality and Education, Tamaulipas Secretariat of Health, and Liaison, United States-Mexico Border Health Commission, Mexico Section, Servicios de Salud de Tamaulipas, Calle Fracisco I

The cornerstone of global public health has been to guarantee and secure good health among all populations. Nonetheless, major barriers to reaching this goal exist. Absence of information is a major barrier to creating adequate health policies and programs and to assessing results of these policies and programs after they have been implemented. We in public health have serious difficulties practicing valid evidence-based public health.

Traditionally, the use of systematically collected data (eg, birth and death data, public health surveillance system data, research and survey data) has been promoted to generate information necessary to manage programs and to create health policies. The general aim is to modify a given disease situation, risk factors, or policies, or to establish policies and strategies that translate into better health for a population. Two major limitations exist to this approach. The first is the internal and external validity of the data, both as a function of the inadequate quality of the data and the tendency to apply the data to a different population from the one in which it was collected. The second is minimal community involvement in the data collection process and in the generation and use of the data.

The approach used in the Brownsville-Matamoros Sister City Project for Women's Health (BMSCP), a surveillance project on reproductive health and medical risk factors conducted in the Mexican municipality of Matamoros, Tamaulipas, and in the United States in Cameron County, Texas, avoided such limitations. Community members participated in analyzing data and generating information. Furthermore, the population generating the information was the same population for which the local health policies and programs that will result from the BMSCP are intended.

A fundamental goal of the BMSCP was to test whether the methods developed for community mobilization, data collection, and information gathering could be useful to health institutions in the US-Mexico border region in making decisions that improve quality of care for women of reproductive age, pregnant women, and their children. Given the project results, we can conclude that such methods can precisely define a health situation and generate the required data to address the local or regional health needs for that situation.

Results of the BMSCP identify specific populations (eg, adolescents) that can benefit from health programs that aim to prevent or decrease health risk. For example, Galván González et al describe how rates of pregnancy and risk of sexually transmitted disease may be high because adolescent girls and young women have low rates of effective contraceptive use (1). Data generated by the BMSCP may allow us to define an integrated and binational approach that considers the physical, behavioral, emotional, and cross-cultural aspects of risk. In another example from the BMSCP, only 34% of women younger than 25 years from the 2 communities used contraceptives during their first sexual encounter (1). This finding suggests that social factors, such as lack of information available through mass media, may have influenced behavior related to the prevention of sexually transmitted infections and pregnancy and makes us question whether the public health strategies we have implemented can be improved. In the state of Tamaulipas in 2007, 37% of births occurred among adolescents (Government of Mexico, Tamaulipas Health Secretariat, unpublished data, 2007). Pregnancy and childbirth exposes adolescents to increased health risks and frequently results in young men and women dropping out of school. Therefore, pregnancy and childbirth in youth is both a public health and societal problem.

Results from the BMSCP related to the initiation of breastfeeding show us the effectiveness of the Mexican strategy to certify "mother-and-child" hospitals that aim not only to provide quality prenatal, delivery, and puerperium care, but also to strengthen the bonding between mother and child. Women who deliver their children in hospitals voluntarily make the decision whether to initiate breastfeeding. Breastfeeding rates in Matamoros and Cameron County are different, which raises questions about the observed differences in the 2 communities where residents have the opportunity to receive care on either side of the border.

BMSCP data from 2005 show that 44% of women overall and in Matamoros had a cesarean delivery (2). Data from the Pumarejo Hospital in Matamoros show that 37% of all deliveries were cesarean, compared with 32% of all deliveries statewide in Tamaulipas (Government of Mexico, Tamaulipas Health Secretariat, unpublished data, 2007). This finding shows that BMSCP study results are concordant with the state information systems but suggests that cesarean delivery may be more common in Matamoros than in Tamaulipas. To better understand these differences, further investigation is needed. However, these results demonstrate that the methods used in the BMSCP raises questions that prompt investigations to get answers, answers that affect health programs and ultimately maternal and child health in our community.

Data from the BMSCP show that only 45% of Matamoros women received prenatal care in the first trimester (2). This finding is consistent with state data, which also indicate that Matamoros women who were pregnant received only 3.5 prenatal care visits on average in 2007 (Government of Mexico, Tamaulipas Health Secretariat, unpublished data, 2007), although the national standard for care is a minimum of 5 prenatal care visits (3). On the basis of these and other examples from the BMSCP provided in this issue of *Preventing Chronic Disease*, the BMSCP method clearly can be used to obtain valid and useful information specific to a population.

The BMSCP experience demonstrates that community participation is critical for the success of community-based surveillance efforts. Early community participation promoted widespread acceptance of and participation in the project, and the methods developed is well suited to the local population. Furthermore, the availability of these data should promote further analysis of the factors that influence reproductive health locally and better use of the information obtained. For public health officials at the local, regional, and state levels, the data generated by the BMSCP method are important for the advancement of the reproductive health in the border population. Having access to a practical method to obtain critical maternal and child health information in the region gives health institutions and other organizations scientific support to explore mechanisms and collaborative approaches that can potentially improve reproductive health in our communities.
